# Supplementary material for: Reconciling Mining with the Conservation of Cave Biodiversity: A Quantitative Baseline to Help Establish Conservation Priorities
Source: PLoS One. 2016 Dec 20;11(12):e0168348. doi: 10.1371/journal.pone.0168348 (PMC5173368; doi:10.1371/journal.pone.0168348)
Supplement: S1 Dataset — (ZIP) [file pone.0168348.s002.zip › Taxa/Serra Norte/SN_2013/N4WS/Lista N4WS-32.pdf]

CAVIDADE N4WS-0032

Classe	Ordem	Fam/Outros	Gên/Outros	Espécie	S-U	U-S	Zon.
Annelida	Oligochaeta			sp.1		3	P
Arachnida	Acari	(Parasitengonina)		sp.5	X	X	P
Arachnida	Acari	(Uropodina)		sp.1	X		P
Arachnida	Acari	Anoetidae		sp.1	X		P
Arachnida	Acari	Argasidae	*Ornithodoros*	sp.1		X	P
Arachnida	Acari	Ascidae		sp.1	X		P
Arachnida	Acari	Macrochelidae		sp.1	X		P
Arachnida	Acari	Trombiculidae		sp.3	X		E
Arachnida	Amblypygi	Charinidae	*Charinus*	jovem		1	P
Arachnida	Araneae	Filistatidae		jovem	X		E
Arachnida	Araneae	Ochyroceratidae	*Ochyrocera*	sp.1		X	P
Arachnida	Araneae	Ochyroceratidae	*Speocera*	sp.1		X	P
Arachnida	Araneae	Ochyroceratidae		jovem	X		E/P
Arachnida	Araneae	Pholcidae	*Leptopholcus*	sp.1	X	X	E/P
Arachnida	Araneae	Tetragnathidae		jovem	X		E
Arachnida	Araneae	Theridiidae	*Achaearanea*	sp.1	X		E
Arachnida	Araneae	Theridiosomatidae	*Plato*	sp.1	X	X	E/P
Arachnida	Opiliones	Cosmetidae		jovem	1		P
Arachnida	Opiliones	Stygnidae	*Protimesius*	*laevis*	1		E
Arachnida	Pseudoscorpiones	Chernetidae	*Spelaeochernes*	sp.1		X	P
Arachnida	Schizomida	Hubbardiidae	aff. *Rowlandius*	sp.1		X	E/P
Entognatha	Collembola	Isotomidae		sp.1		X	P
Entognatha	Collembola	Paronellidae		sp.1	X	X	E
Entognatha	Collembola	Paronellidae		sp.4		X	P
Entognatha	Diplura	Campodeidae		sp.1		X	E/P
Insecta	Blattodea	Blaberidae		jovem		1	P
Insecta	Blattodea	Polyphagidae		jovem		2	E
Insecta	Blattodea			jovem	1		P
Insecta	Coleoptera	Dytiscidae		sp.11B	X		P
Insecta	Coleoptera	Elminthidae		jovem	X		P
Insecta	Diptera	Ceratopogonidae		sp.	X		E
Insecta	Diptera	Dolichopodidae		sp.	X		E
Insecta	Diptera	Drosophilidae	*Drosophila*	*eleonorae*	X		E
Insecta	Diptera	Muscidae		jovem	X		P
Insecta	Diptera	Psychodidae	*Sciopemyia*	*sordellii*	X	X	E/P
Insecta	Diptera	Tipulidae	Tipulinae	sp.	X		E
Insecta	Heteroptera	Schizopteridae	Schizopterinae	sp.1		X	P
Insecta	Homoptera	Cercopidae		jovem		X	P
Insecta	Homoptera	Cixiidae		jovem	X		P
Insecta	Hymenoptera	Formicidae	*Camponotus*	*atriceps*		1	P
Insecta	Hymenoptera	Formicidae	*Camponotus*	sp.1	X	X	P
Insecta	Hymenoptera	Formicidae	*Gnamptogenys*	*striatula*	X		E
Insecta	Hymenoptera	Formicidae	*Hypoponera*	sp.1	X		P
Insecta	Hymenoptera	Formicidae	*Nylanderia*	sp.2	X		E
Insecta	Hymenoptera	Formicidae	*Octostruma*	sp.1	X		E
Insecta	Hymenoptera	Formicidae	*Strumigenys*	sp.1		X	P
Insecta	Hymenoptera	Formicidae	*Wasmannia*	sp.1		X	P
Insecta	Isoptera	Termitidae	*Nasutitermes*	sp.	X		P
Insecta	Lepidoptera	Noctuoidea		sp.1	2	8	E

Insecta	Lepidoptera	Noctuoidea		sp.2		X	E
Insecta	Lepidoptera	Noctuoidea		sp.4		X	E
Insecta	Orthoptera	Phalangopsidae	*Paraclodes*	sp.	3	1	E
Insecta	Orthoptera	Phalangopsidae	*Phalangopsis*	sp.	16	31	E/P
Insecta	Psocoptera	Psyllipsocidae	*Psyllipsocus*	sp.3		X	E
Turbellaria	Tricladida			sp.1	X	X	P
Amphibia	Anura	Strabomantidae	*Pristimantis*	cf. *fenestratus*	10	2	E/P
Mammalia	Chiroptera	Emballonuridae	*Peropteryx*	sp.	3	2	P
